# Resistance of Uropathogens to Tebipenem: An Analysis of the Evidence from In Vitro Antimicrobial Susceptibility Studies

**DOI:** 10.3390/microorganisms14030726

**Published:** 2026-03-23

**Authors:** Matthew E. Falagas, Christina-Maria Asimotou, Dimitrios S. Kontogiannis, Laura T. Romanos, Panagiota Poziou, Iva D. Tzvetanova

**Affiliations:** 1 Alfa Institute of Biomedical Sciences, 15123 Athens, Greece; 2 School of Medicine, European University Cyprus, 2404 Nicosia, Cyprus; i.tzvetanova@euc.ac.cy; 3 Department of Medicine, Tufts University School of Medicine, Boston, MA 02111, USA

**Keywords:** antimicrobial therapy, carbapenem, *E. coli*, *E. faecalis*, Enterobacterales, *K. pneumoniae*, multidrug-resistant, penicillin-binding protein, *P. mirabilis*, tebipenem

## Abstract

Tebipenem is a new carbapenem antibiotic that binds to penicillin-binding proteins (PBPs). Given the need for effective antibiotics against multidrug-resistant (MDR) bacteria, this review evaluated the in vitro antimicrobial activity of tebipenem against Gram-negative and Gram-positive bacteria, focusing on uropathogens. Five resources (Google Scholar, Web of Science, Embase, Scopus, and PubMed) were used to identify relevant articles. Of the 1322 articles identified, 9 relevant studies were included, which evaluated 12,501 Gram-negative and 122 Gram-positive pathogens. All nine studies (100%) assessed the activity of tebipenem against *Escherichia coli*, with an MIC_90_ value range of 0.015–>4 mg/L. Seven studies (77.8%) included *Klebsiella pneumoniae*, with an MIC_90_ value range of 0.015–0.5 mg/L. Six studies (66.7%) reported data on *Proteus mirabilis*, with an MIC_90_ value range of ≤0.125–0.5 mg/L. Two studies (22.2%) evaluated the activity of tebipenem against *Enterococcus faecalis*, with MIC_90_ of 1 mg/L among vancomycin-susceptible isolates and 32 mg/L in isolates with not-reported mechanisms of resistance. Two studies (22.2%) evaluated the activity of tebipenem against *Enterococcus faecium*, with MIC_90_ of >4 mg/L among both vancomycin-susceptible and vancomycin-resistant isolates and MIC_90_ of 128 mg/L among isolates with no resistance mechanism reported. Tebipenem demonstrated good activity against Enterobacterales, such as *E. coli* and *K. pneumoniae*. The antimicrobial agent exhibited higher MICs and a higher proportion of resistance among *P. mirabilis* isolates. Tebipenem could be effective for outpatient treatment of infections caused by MDR Gram-negative pathogens. However, given its potential to exert selective pressure for the development of antimicrobial resistance, it should be considered for patients with cUTIs when none of the first-line treatment options demonstrate in vitro antimicrobial activity.

## 1. Introduction

Tebipenem is a novel member of the carbapenem subclass of β-lactam antibiotics. Like other β-lactams, it binds strongly to penicillin-binding proteins (PBPs) and therefore inhibits the synthesis of the bacterial cell wall (inhibition of peptidoglycan synthesis) [1,2]. Structural studies of the PBP–carbapenem complexes in *Streptococcus pneumoniae* demonstrate that the carbapenem C-2 side chains of tebipenem interact intimately with Trp374 and Thr526 of PBP 2X and with Trp411 and Thr543 of PBP 1A, forming hydrophobic interactions. This strong binding is the key pharmacologic feature of tebipenem’s mechanism of action [2,3,4].

Chemically, a nitrogen heterocyclic group on the C3 side chain of tebipenem pivoxil forms a prodrug by interacting with C2 carboxylic acids, significantly elevating oral absorption, which plays a key role in the higher antibacterial activity of tebipenem pivoxil compared with most other β-lactam antibiotics [1]. The prodrug form, tebipenem pivoxil (C_22_H_31_N_3_O_6_S_2_, ~497.63 g/mol), is designed to enhance intestinal absorption and bioavailability by virtue of its esterified form, which is then converted by esterases in the gut to the active drug [5,6]. The active moiety (tebipenem) has the molecular formula C_16_H_21_N_3_O_4_S_2_ and a molecular weight of approximately 383.49 g/mol [7,8]. The mechanism of enhanced absorption is further supported by transporter-mediated uptake, as proved by “Caco-2” cell experiments. These experiments use a cell line derived from human colorectal adenocarcinoma, which, when cultured under the right conditions, differentiates and acquires many features of small-intestinal absorptive (enterocyte) cells. It is therefore widely used as an in vitro model of the human intestinal epithelium to study absorption, transport, metabolism, barrier function, and toxicity. The results of these experiments showed that tebipenem pivoxil uptake was decreased by adenosine triphosphate (ATP) depletion and temperature lowering, and significant transport activity via human OATP1A2 (Km ≈ 41.1 µM) and OATP2B1 (Km > 1 mM) was demonstrated, indicating that multiple intestinal influx transporters contribute to its high absorptive profile [6].

Tebipenem was first developed by Pfizer Inc. (New York, NY, USA). Its granule preparation, a product of Meiji Inc. (Tokyo, Japan), was approved and listed in April 2009 under the trade name “Orapenem” for pediatric otorhinolaryngological infections, including otitis media, sinusitis, and pneumonia [1,9]. Worldwide development [for adult indications such as complicated urinary tract infection (cUTI) and acute pyelonephritis (AP)] is currently underway, with an oral tebipenem pivoxil hydrobromide formulation as the focus [10]. In the broader development context, tebipenem showed promising in vitro activity against Enterobacterales, including extended-spectrum β-lactamase (ESBL)- and AmpC-producing strains. However, it remains vulnerable to hydrolysis by certain carbapenemases [e.g., *Klebsiella pneumoniae* carbapenemase (KPC), oxacillinase-48 (OXA-48), New Delhi metallo-β-lactamase-1 (NDM-1)] produced by this bacterial category [11,12]. Interestingly, during the “PIVOT-PO” Phase 3 trial (2024–2025), in which hospitalized adults with cUTI or AP were randomized to oral tebipenem hydrobromide vs. intravenous imipenem–cilastatin, an independent committee stopped the trial early for efficacy after the primary endpoint (non-inferiority) was met in May 2025. No new safety concerns were noted, and “GlaxoSmithKline” indicated plans to file with the US Food and Drug Administration (FDA) in the second half of 2025 [13].

As an orally bioavailable agent with a broad spectrum of activity, including activity against resistant Gram-negative Enterobacterales, tebipenem addresses a critical gap in the treatment options for patients with infections caused by multidrug-resistant (MDR) bacteria. It is a viable oral alternative for outpatient management and facilitating an earlier step-down therapy. In this context, we aimed to review data on the resistance of Gram-negative and Gram-positive bacteria to this novel oral carbapenem and to identify its potential strengths and weaknesses.

## 2. Literature Search

For the identification, screening, and inclusion of relevant articles, five resources were used (Google Scholar, Web of Science, Embase, Scopus, and PubMed) from inception to October 2025. A search strategy with the keywords “tebipenem,” “SPR994,” “in vitro,” “susceptibility,” resistance,” and “minimum inhibitory concentration” was used. The detailed search strategy is presented in Supplementary Table S1.

Inclusion criteria for the reviewed studies were: (a) primary research articles, (b) studies that included tebipenem or SPR994 in the title/abstract/keywords, and (c) studies that reported MIC ranges, MIC_50_, MIC_90_, or susceptibility proportions (%) for Gram-negative or Gram-positive pathogens to tebipenem that cause UTIs. Studies were excluded if (a) they were non-primary research articles, (b) evaluated specific β-lactamase-harboring isolates, (c) did not include specific data on tebipenem, or (d) assessed five or fewer isolates.

Two independent reviewers (C.M.A. and D.S.K.) extracted data from the included studies and tabulated them. Disagreements between reviewers were resolved by consensus with a senior author (M.E.F.). Details of the included studies, including country, study type (single-center, multi-center, or surveillance), and isolation sources, were tabulated. Additionally, the total number of isolates, along with the resistance mechanism, MIC range, MIC_50_, MIC_90_, and proportions of intermediate resistance or resistance, were extracted.

### 2.1. Evaluation of Antimicrobial Resistance

The proportions of intermediate resistance and resistance among Gram-negative and Gram-positive bacteria to tebipenem were presented in a table alongside the MIC range, MIC_50_, and MIC_90_. For studies that provided relevant data, proportions were given according to the antimicrobial susceptibility breakpoints specified in each study. Since there are no clinical breakpoints suggested by EUCAST or CLSI, the breakpoints used to support in vitro data or clinical trials are preliminary and were set by each laboratory. In cases where a study provided data on the proportion of susceptible isolates without information on the proportions of isolates with intermediate resistance or resistance, the proportion of non-susceptible isolates was reported.

### 2.2. Resistance to Tebipenem

**Figure 1:**
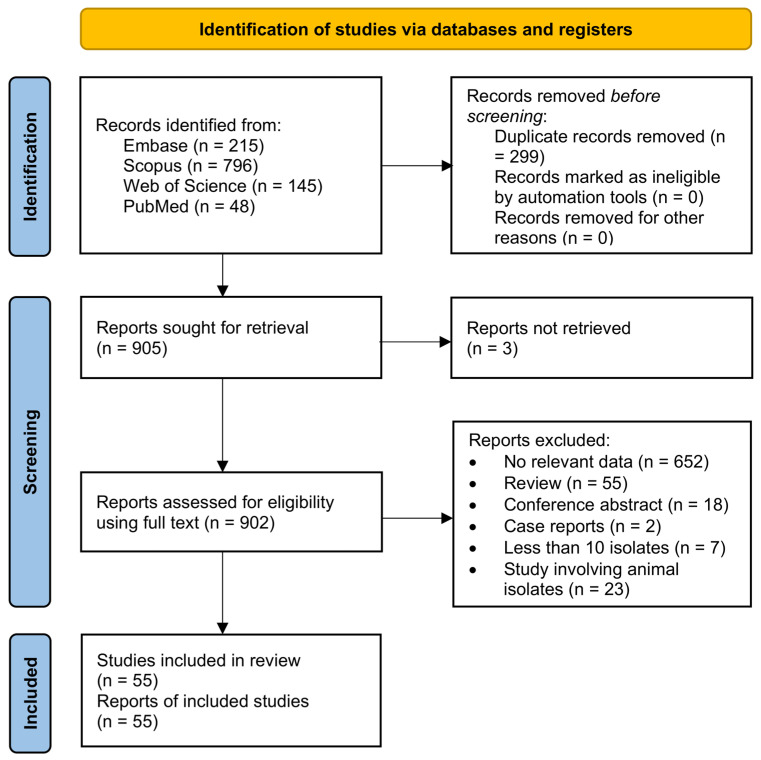
Flow diagram for the evaluation, selection, and inclusion of relevant articles.

Figure 1 presents the flow diagram of identification, screening, and inclusion of relevant articles. In total, 1322 articles were identified from the five resources (Google Scholar, Web of Science, Embase, Scopus, and PubMed). After duplication, 164 were excluded, and 1158 were assessed based on title and/or abstract. After excluding 1131 articles, 27 remained for full-text evaluation. Eighteen articles were excluded: 15 were conference abstracts, two did not provide specific antimicrobial susceptibility data for tebipenem, and one study evaluated specific β-lactamases in *Escherichia coli* isolates. In total, nine articles were included in this review [1,14,15,16,17,18,19,20,21].

Table 1 presents data for Gram-negative bacterial isolates regarding the country of pathogen isolation, the year of publication, the type of study (multi-center or single-center), source of isolation of pathogens (or type of infection from where pathogens were collected), the pathogens evaluated, the various resistance mechanisms (when assessed) and the MIC_50_, MIC_90_, and MIC range of the nine included studies. In total, eight were multi-center studies [1,14,15,16,18,19,20,21], and one was a single-center study [17]. The studies included a total of 12,501 isolates.

Nine studies (100%) evaluated the activity of tebipenem against *E. coli* isolates [1,14,15,16,17,18,19,20,21]. The *E. coli* isolates harbored different resistance genes. The results showed that 1040 of the isolated *E. coli* harbored ESBL-producing mechanisms [17,18,19,20,21], 4284 were non-ESBL-producing pathogens [17,18,20], 10 were both MDR and ESBL-producing bacteria [17], 24 were pAmpC-positive [21], 4 were both pAmpC- and ESBL-producing [21], and for 790 isolates, no mechanism of resistance (NR) was reported [1,14,15,16]. According to these studies, the MIC_90_ for the isolates ranged from 0.015 mg/L to >4 mg/L [1,14,15,16,17,18,19,20,21]. Two studies (22.2%) included an *Enterobacterales* category that contained the main species isolates of the studies (*E. coli, K. pneumoniae, P. mirabilis*) along with isolates of other species [15,20], and had MIC_90_ of 0.125 mg/L and 0.06 mg/L, respectively.

Seven studies (77.8%) [1,14,15,16,17,20,21] evaluated the resistance mechanisms and MICs of *K. pneumoniae* to tebipenem. The *K. pneumoniae* isolates harbored different resistance genes. The results showed that out of all *K. pneumoniae* isolates (1082), 149 were ESBL-producing [17,20,21], 465 were non-ESBL-producing pathogens [17,20], 2 were both ESBL- and pAmpC-producing [21], one was only expressing the pAmpC gene [21], and for 459 *K. pneumoniae* isolates, no mechanism of resistance (NR) was reported [1,14,15,16]. MIC_90_ value of these isolates ranged from 0.015 mg/L to 0.5 mg/L [1,14,15,16,17,20,21]. The ESBL-producing *K. pneumoniae* isolates, those producing both ESBL and pAmpC, and the one isolate producing only pAmpC, presented higher resistance to tebipenem (MIC_90_ of 0.125 mg/L in two studies [17,21] and 0.03 mg/L in one study [20]) than the non-ESBL-producing isolates (MIC_90_ of 0.015 mg/L) [17]. Also, studies that did not report the mechanism of resistance presented MIC_90_ values of 0.06 mg/L [14,15], 0.25 mg/L [16], and 0.5 mg/L [14].

Additionally, six studies (66.7%) [1,14,15,16,17,20] evaluated the resistance mechanisms and MICs of *Proteus mirabilis* to tebipenem. The *P. mirabilis* isolates harbored different resistance genes. Of 473 *P. mirabilis* isolates, 10 were ESBL-producing [20], 225 were non-ESBL-producing [20], and the rest (238) were not reported (NR) as having any resistance mechanism [1,14,15,16,17]. The maximum MIC_90_ ranged from ≤0.125 mg/L to 0.5 mg/L [1,14,15,16,17,20].

Table 2 presents data about Gram-positive bacteria regarding the country of pathogen isolation, the year of publication, the type of study (multi-center [1] or single-center [17]), source of isolation of pathogens (or type of infection from where pathogens were collected), the pathogens evaluated, the various resistance mechanisms (when assessed) and the MIC_50_, MIC_90_, and MIC range of the two included studies.

Both studies (100%) evaluate the resistance mechanism and MICs of *Enterococcus faecalis* and *E. faecium* to tebipenem. Of 45 *E. faecalis* isolates, 35 were susceptible to vancomycin [17], while the remaining 10 showed no reported resistance mechanisms [1]. Notably, the MIC_90_ for the vancomycin-susceptible isolates was 1 mg/L [17], and for the rest (NR mechanism of resistance) it was 32 mg/L [1]. Of 65 *E. faecium* isolates, 35 were vancomycin-susceptible [17], and 20 were resistant to vancomycin [17]. At the same time, the remaining 10 showed no reported resistance mechanisms [1]. Notably, the MIC_90_ for both the vancomycin-susceptible and vancomycin-resistant isolates was >4 mg/L [17] and for the rest (NR mechanism of resistance) it was 128 mg/L. Only one study (50%) [17] evaluated the MIC of Group B β-hemolytic *Streptococci* against tebipenem, with the MIC_90_ being 0.015 mg/L.

### 2.3. Evaluation of the Published Evidence

Overall, across the included studies, tebipenem demonstrated low MIC_50_ and MIC_90_ values against most Enterobacterales isolates, indicating potent antibacterial activity. Resistance, especially among *E. coli* and *K. pneumoniae*, was generally rare, although resistance varied depending on the presence of resistance mechanisms.

In evaluating *E. coli* results, tebipenem showed very good activity against the pathogen across most studies. Resistance proportions were low, highlighting the potential utility of tebipenem against resistant *E. coli* strains.

Tebipenem showed good antimicrobial activity against *K. pneumoniae* in the included studies. *K. pneumoniae* may have reduced susceptibility to tebipenem compared to *E. coli*. This can be observed in two of the included studies, where MICs ranged from >32 mg/L and >8 mg/L, respectively. No resistance mechanism was reported for the *K. pneumoniae* isolates in one of the aforementioned studies [14], whereas in the other study [20], 81 *K. pneumoniae* isolates out of a total 511 were ESBL-producing pathogens. Still, the drug’s activity remains efficient, as can be seen from the rest of the studies. Very little data on the resistance of both ESBL- and pAmpC- strains are available, suggesting that further research is needed for isolates harboring these resistance mechanisms.

In contrast to *E. coli* and *K. pneumoniae*, *P. mirabilis* exhibited higher MICs and higher proportions of resistance in the analyzed studies. This pattern suggests that the activity of tebipenem against *P. mirabilis* is moderate, according to the available data, possibly due to intrinsic or acquired resistance mechanisms of isolates of this species. However, further research on this pathogen is needed for more robust conclusions.

Comparison of tebipenem MIC distributions across geographic regions suggests that species-specific variability is more pronounced than true regional resistance divergence, although some heterogeneity is evident. Large multicenter UTI surveillance studies from the USA (Mendes 2022, 2023, Asempa 2023) [15,19,20] and Japan (Ito 2025) [18] consistently report very low MIC values for *E. coli*, including ESBL-producing isolates, indicating stable activity across these regions. Similarly, European and multinational datasets (Arends 2019, Ranasinghe 2022) [14,21] show comparable MIC ranges for *E. coli* and *K. pneumoniae*, supporting geographic reproducibility of activity against these species. In contrast, greater variability is observed for *K. pneumoniae* and particularly *P. mirabilis*, with U.S.-based cohorts demonstrating higher MIC_90_ values and increased proportions of intermediate-resistance or largely resistant isolates, suggesting that local resistance and clonal distribution may influence susceptibility patterns. The Chinese study by Yao (2016) [1] also reported broader MIC dispersion for certain species and markedly high MICs for *P. aeruginosa*. This information may suggest that tebipenem’s limited activity is consistent across regions rather than geographically driven. Overall, the data indicate that while tebipenem maintains stable potency against *E. coli* globally, interspecies differences account for most observed MIC variability, with local resistance mechanisms potentially amplifying these differences in certain settings.

Gram-positive bacteria demonstrate generally low tebipenem MIC values, although variability between studies is evident. In the study by Gerges (2023) [17], tebipenem showed strong in vitro activity against vancomycin-susceptible *E. faecalis* isolates (MIC_50_ 0.25 mg/L, MIC_90_ 1 mg/L), as well as very good activity against Group B β-hemolytic streptococci (MIC_50_ ≤ 0.004 mg/L, MIC_90_ 0.015 mg/L). However, the in vitro activity of tebipenem was weaker (>4 mg/L MIC_90_ for both vancomycin-susceptible and vancomycin-resistant *E. faecium* isolates, indicating a wide MIC distribution between the different species’ isolates of this single-center cohort. The findings of these 2 studies appear to indicate that *E. faecium* isolates, regardless of their susceptibility to vancomycin, are less susceptible than *E. faecalis* to tebipenem. In contrast, the multicenter study by Yao (2016) [1] reported a similar MIC_50_ for *E. faecalis* (0.25 mg/L) but a markedly elevated MIC_90_ (32 mg/L). This suggests greater heterogeneity in susceptibility and the possible presence of less susceptible subpopulations, although such a conclusion is not safely precluded due to lack of further studies available. The findings of the same study for *E. faecium* indicate a much higher MIC_90_ of 128 mg/L, though with no resistance mechanism reported [1]. The absence of detailed resistance mechanism data in the Yao study [1] limits interpretation, underlining the importance of continued surveillance and standardized susceptibility reporting for Gram-positive bacteria. However, the findings of these 2 studies concerning the in vitro activity of tebipenem against *E. faecium* isolates, regardless of their susceptibility to vancomycin, suggest that this pathogen may be less susceptible than *E. faecalis* to tebipenem.

### 2.4. Mechanism of Action and Resistance

Tebipenem has a mechanism of action similar to other carbapenems; however, its potent activity and promising efficacy is supported by the fact that it is absorbed as a pro-drug form, which enhances its bioavailability, and it is afterwards converted to its active drug form by the intestine’s esterases. It is an orally available carbapenem antibiotic that binds strongly to PBPs and therefore inhibits the synthesis of the peptidoglycan, and thus the bacterial cell. After the absorption of the pro-drug (tebipenem pivoxil) and its conversion to the active form, tebipenem penetrates the bacterial periplasm (in Gram-negative bacteria) and binds strongly to PBPs (enzymes which catalyze the transpeptidation step of peptidoglycan cross-linking in the bacterial cell wall) [22,23].

Especially in Gram-negative bacteria, tebipenem shows a potent inhibition of PBP-2 (and multiple PBPs in Gram-positives). Thus, tebipenem prevents cross-linking of the peptidoglycan strands, weakening the cell wall architecture and resulting ultimately in the lysis of the bacterium [22,24]. Structural or crystallographic work (in PBP2X and PBP1A *Streptococcus pneumoniae* complexes) shows that tebipenem forms a covalent acyl-enzyme adduct stabilized by specific hydrophobic and hydrogen-bond interactions between tebipenem’s C-2 side chain and conserved tryptophan and threonine residues (specifically the Trp374 and Thr526 residues in PBP 2X and the Trp411 and Thr543 residues in PBP 1A) [2,3,4]. This formation is the key to tebipenem’s tight PBP engagement. These biochemical and microbiological findings are supported by in vitro and structural studies that identify tebipenem as a potent multi-PBP inhibitor with a preference for PBP-2 in Gram-negative bacteria [22,24].

In addition to its PBP-binding mechanism, tebipenem has structural features that confer relative stability against many β-lactamases. In a pharmacokinetic biochemical study with the β-lactamase *bla*_C_ from *Mycobacterium tuberculosis*, tebipenem was shown to form a very stable acyl-enzyme adduct (with kₘ ≈ 0.8 µM and kₐₜ ≈ 0.03 min^−1^), which persisted for several minutes. Therefore, it inhibited the enzyme rather than being rapidly hydrolyzed [25,26]. This explains both the enzyme inhibition and the relative stability against many ESBL and AmpC enzymes. The carbapenem core of tebipenem (mainly the 6-hydroxyethyl substituent and 1β-methyl group) helps hinder access of β-lactamases to the β-lactam ring. It contributes to its broad spectrum of activity against ESBL and AmpC-producing Enterobacterales. However, carbapenemases such as *bla*_KPC_, *bla*_NDM_, *bla*_VIM_, *bla*_IMP_, and *bla*_OXA-48_ can efficiently hydrolyze tebipenem. Studies show that these enzymes have high catalytic efficiency for tebipenem, similar to other carbapenems such as meropenem or imipenem. Once hydrolyzed, tebipenem loses its ability to bind to PBPs and therefore becomes inactive [11,14,27]. Although considering that CTX-M and AmpC β-lactamases represent the primary determinants of multidrug-resistant cUTIs, the stability of tebipenem to hydrolysis by these enzymes supports the utility of its prodrug, tebipenem pivoxil hydrobromide, as an oral therapy for adult cUTIs [11].

It is important to note that none of the pathogens included in our study appeared to produce carbapenemases. A subset of pathogens was ESBL- or pAmpC-producing, or resistant to levofloxacin and cotrimoxazole, but did not produce any carbapenemases [such as KPC, NDM, Verona integron-encoded metallo-β-lactamase (VIM), imipenemase (IMP), OXA-48].

### 2.5. Effectiveness and Safety of Tebipenem

Studies testing the effectiveness and safety of tebipenem have been conducted since before the 2000s. The first clinical studies on tebipenem were Japanese pediatric trials in the 1990s, in which the drug was used to treat common otolaryngological and upper respiratory tract infections [1]. After these studies highlighted the drug’s effectiveness in pediatric patients, its use began in April 2009 for otorhinolaryngological infections in this patient population.

The first adult trial of tebipenem pivoxil hydrobromide, the Japanese Phase 3 trial in adult patients with cUTI, was conducted in 2020. This study evaluated the efficacy of oral tebipenem pivoxil hydrobromide compared with IV ertapenem in 661 patients with cUTI or AP. The mean age of the patients who participated was 56 years, about 70% were female, and key comorbidities included diabetes (approximately in 30% of the participants) and urinary tract structural disease (approximately in 20%). Pathogens mirrored the global data, with *E. coli* (82%) and *Klebsiella* spp. (9%) being the most common causative pathogens. Additionally, ESBL-positive isolates were present in about one-quarter of patients [28].

The second trial about tebipebem’s effectiveness and safety was the “ADAPT-PO” Phase 3 trial (NCT03788967). It was a randomized, multicenter, multinational, double-blind, double-dummy, noninferiority study. The patients enrolled represented different regions (USA, Eastern Europe, Russia, South Africa) [29]. It consisted of two arms. One received oral tebipenem pivoxil hydrobromide (600 mg every 8 h), while the second received intravenous ertapenem (1 g daily). The total number of participants was 1372 hospitalized adults with complicated cUTIs or AP [29,30,31,32]. The study population included adults aged 18–93 years (mean age 55 years). 87% were women, and approximately 26% had diabetes mellitus, data that are representative of typical cUTI patients. Most infections were caused by *E. coli* (in 81% of the patients), with ESBL-producing Enterobacterales being identified in approximately 24% of isolates [29]. Patients received treatment for 7–10 days (up to 14 days for bacteremia). They were evaluated for the composite primary endpoint of overall response, both clinical cure and microbiologic eradication, at the test-of-cure visit (day 19 ± 2). The overall response was 58.8% and 61.6% with tebipenem and ertapenem, respectively, meeting the prespecified non-inferiority margin (difference of 3.3 percentage points, 95% CI: −9.7 to 3.2) [31,32,33]. Clinical cure occurred in over 93% of patients in both arms. Microbiologic eradication rates were similar between groups, and safety was comparable. Treatment-emergent adverse events occurred in 25.7% of patients receiving tebipenem pivoxil hydrobromide and 25.6% of patients receiving ertapenem. Most adverse events were mild (diarrhea, nearly 5%; headache, nearly 3.8%), with very rare (less than 2%) serious adverse events and no deaths reported [33].

The pivotal “PIVOT-PO” Phase 3 trial (NCT06059846) is the largest-scale study as of 2025. It was conducted between 2023 and 2025. It enrolled hospitalized adults with cUTIs or AP to compare oral tebipenem hydrobromide 600 mg every 8 h with IV imipenem–cilastatin (500 mg of imipenem and 500 mg of cilastatin) every 6 h [13]. Approximately 1690 patients were included in the interim efficacy analysis, and represented North America, Europe, Latin America, and the Asia-Pacific region. Eligible participants were adults (18 years and older) with confirmed cUTI or AP and a baseline urine culture demonstrating >105 CFU/mL of a uropathogen. The most common pathogens were E. coli (approximately 80%), K. pneumoniae (10–15%), P. mirabilis, and Enterobacter cloacae. Roughly a quarter of isolates produced ESBLs [34,35]. The mean patient age was approximately 54 years, and approximately 65% were females. Common comorbidities reflected the typical cUTI profile: diabetes mellitus (20–25% of the studied population), chronic kidney disease stages II–III (about 15%), and structural urinary tract abnormalities or retention [13]. Roughly 45% of participants had AP, while the rest of the participants presented with cUTI without pyelonephritis. Patients with creatinine clearance of more than 30 mL/min were included [36]. Dosing was adjusted for moderate renal impairment [37]. To ensure controlled infection management and safety monitoring, all participants were hospitalized at study entry [38]. Although the trial stopped early for efficacy in May 2025, it met the primary endpoint of non-inferiority of tebipenem hydrobromide compared to intravenous imipenem–cilastatin in hospitalized adult patients with cUTIs, including pyelonephritis, on overall response (composite of clinical cure plus microbiological eradication) at the test-of-cure visit [13].

### 2.6. Role of Tebipenem in Clinical Practice

Tebipenem is a valuable addition to the management of patients with infections due to Gram-negative pathogens, including strains that produce ESBLs and certain quinolone-resistant strains. However, it presents a spectrum constraint because it lacks clinically sufficient activity against organisms such as *P. aeruginosa* (like other carbapenems and penems, specifically ertapenem and sulopenem, respectively), *P. mirabilis*, and Group B β-hemolytic streptococci. Therefore, tebipenem’s role in the antibiotic strategy should be reserved for targeted therapy and not for empirical treatment of severe hospital-acquired infections or infections in patients at high risk for *P. aeruginosa* (e.g., patients with cystic fibrosis, neutropenic patients, and burn victims). Administration should be reserved exclusively for cases in which antimicrobial susceptibility testing (antibiogram) confirms that the isolate is susceptible to tebipenem and not to any of the first-line antibiotic choices [39]. One study on tebipenem as a step-down therapy after intravenous ertapenem showed that transitioning from intravenous ertapenem to oral tebipenem reduced hospital length of stay, nosocomial infection risk, and costs, and improved patient satisfaction. This data demonstrates the potential role of tebipenem as an oral transition agent from intravenous antibiotic regimens within the antibiotic stewardship paradigm, once tebipenem susceptibility is confirmed by antibiogram [40]. However, further research is needed to preclude a safe conclusion [40]. Additionally, patients should only be switched from intravenous to oral antibiotic therapy when they are hemodynamically stable, improving clinically, able to ingest medications, and have a normally functioning gastrointestinal tract [41].

Additionally, oral administration of an antibiotic with a broad-spectrum antimicrobial activity, such as tebipenem, may exert significant selective pressure on the intestinal microbiota, a major reservoir of Enterobacterales. This pressure suppresses susceptible organisms while favoring the persistence and expansion of bacteria harboring various antimicrobial resistance genes, including carbapenemase genes (e.g., KPC, NDM), thereby facilitating intestinal colonization and subsequent dissemination of carbapenem-resistant organisms within the community and healthcare settings [39].

This information suggests that, despite its very promising role in facing many Gram-negative and Gram-positive bacteria, more research should be done in order to clarify major undefined topics concerning tebipenem’s role and position in antimicrobial therapy. Although the pivotal “PIVOT-PO” Phase 3 trial (NCT06059846) has demonstrated non-inferiority for complicated urinary tract infections [13], additional confirmatory analyses and real-world effectiveness studies are needed to better define optimal patient populations, including subgroups such as those with pyelonephritis or prior antimicrobial exposure. Given the significance of carbapenems in antimicrobial stewardship, long-term surveillance studies assessing tebipenem resistance selection and shifts in tebipenem susceptibility patterns will be critical. Further investigation into the drug’s impact on the intestinal microbiome, particularly in vulnerable populations, is also warranted to evaluate risks related to colonization with multidrug-resistant organisms. From a pharmacological perspective, continued refinement of pharmacokinetic and pharmacodynamic modeling—especially in special populations such as patients with renal impairment, extremes of age, or complex comorbidities—will help optimize dosing strategies. Finally, implementation studies exploring oral step-down strategies, hospital length of stay and readmission rates, will be essential to determine tebipenem’s practical value within antimicrobial stewardship frameworks.

### 2.7. Limitations

Our review is not without limitations. Although the evaluation of the available literature was thorough, the available data for Gram-positive cocci were limited. Also, several studies did not report susceptibility percentages of the studied isolates to tebipenem. Moreover, the available tebipenem breakpoints involved Gram-negative bacteria (*E. coli*, *K. pneumoniae*, *P. mirabilis*, *Enterobacterales*, *Citrobacter* spp., *Enterobacter aerogenes*, *E. cloacae*, *and Pseudomonas aeruginosa*), but not Gram-positive bacteria (*E. faecalis*, *E. faecium*, and Group B β-hemolytic streptococci). Consequently, analysis of the resistance percentages of bacterial isolates from some species was not possible.

## 3. Conclusions

Tebipenem represents a promising orally administered option for the treatment of complicated urinary tract infections (cUTIs), particularly those caused by Enterobacterales. Surveillance data suggest that interspecies differences in tebipenem susceptibility appear more consistent than clear regional divergence. Data suggests that it has consistently good activity against *E. coli*, including ESBL-producing isolates. Activity against *K. pneumoniae* is also generally favorable, though with slightly higher and more variable MIC distributions, while Proteus mirabilis exhibits comparatively elevated MIC values and higher proportions of intermediate or resistant isolates in some cohorts. Importantly, the available surveillance data demonstrate limited activity against Pseudomonas aeruginosa, reinforcing tebipenem’s species-specific spectrum. The data available for Gram-positive bacteria is no indication for a safely precluded conclusion, as the studies dedicated to these bacteria are very scarce. The lack of available data highlights the importance of ongoing resistance monitoring. Given the ecological significance of carbapenems, tebipenem should be reserved for cUTI cases in which first-line agents lack in vitro activity, and its clinical implementation should be accompanied by structured antimicrobial stewardship programs and continuous regional susceptibility surveillance to mitigate the risk of community-level carbapenem resistance emergence.

## Figures and Tables

**Table 1:** Resistance of Gram-negative bacterial isolates to tebipenem.

Author	Year	Country	Single- or Multi-Center (Surveillance)	Sources of Isolation (Infection or Body Site)	Isolates	N	Resistance Mechanism, *n*	MIC Range	MIC_50_	MIC_90_	**I**	**R**
								mg/L			***n*** **(%)**	
Arends [14]	2019	Europe ^a^, Israel, Turkey, USA	Multi-center	UTIs	*E. coli*	101	NR	≤0.015–0.12	≤0.015	0.03	NR	NR
					*K. pneumoniae*	208	NR	≤0.015–>32	0.03	0.06	NR	NR
					*P. mirabilis*	103	NR	0.03–0.5	0.06	0.12	NR	NR
Asempa [15]	2023	USA	Multi-center	519 urine, 74 wound/soft tissue, 25 other ^b^	Enterobacterales	618	161 ESBL phenotype, 95 levofloxacin and cotrimoxazole-R,172 MDR	≤0.004–>4	0.016	0.125	45 (7.3) ^c^	15 (2.4) ^c^
					*E. coli*	362	NR	≤0.004–>0.25	0.016	0.125	3 (0.8) ^c^	0 (0) ^c^
					*K. pneumoniae*	92	NR	0.008–>4	0.03	0.06	3 (3.2) ^c^	2 (2.2) ^c^
					*P. mirabilis*	63	NR	0.016–4	0.125	0.5	24 (38.1) ^c^	7 (11.1) ^c^
Fouad [16]	2025	USA	Multi-center	NR	*All*	500	NR	NR	0.02	0.25	38 (7.6)	17 (3.4)
					*E. coli*	343	NR	NR	0.02	0.03	7 (2) ^c^	0 (0) ^c^
					*K. pneumoniae*	79	NR	NR	0.03	0.25	5 (6.3) ^c^	4 (5.1) ^c^
					*P. mirabilis*	37	NR	NR	0.25	0.5	18 (48.7) ^c^	8 (21.6) ^c^
Gerges [17]	2023	USA	Single-center	Blood cultures	*Citrobacter* spp.	15	NR	≤0.004–0.06	0.015	0.06	NR	0 (0) ^d^
					*E. aerogenes*	21	NR	0.008–0.5	0.03	0.06	NR	1 (4.8) ^d^
					*E. cloacae*	30	NR	0.008–0.125	0.03	0.125	NR	0 (0) ^d^
					*E. coli*	33	ESBL-prod.	0.008–0.125	0.015	0.03	NR	0 (0) ^d^
					*E. coli*	32	Non-ESBL-prod.	≤0.004–0.03	0.008	0.015	NR	0 (0) ^d^
					*E. coli*	10	MDR and ESBL-prod.	0.015–>4	0.125	>4	NR	NR
					*K. pneumoniae*	35	ESBL-prod.	0.008–0.25	0.03	0.125	NR	1 (2.9) ^d^
					*K. pneumoniae*	35	Non-ESBL-prod.	0.008–0.03	0.015	0.015	NR	0 (0) ^d^
					*P. mirabilis*	20	NR	0.015–0.125	0.06	0.125	NR	0 (0) ^d^
Ito [18]	2025	Japan	Multi-center	Community-acquired complicated UTIs	*E. coli*	290	61 ESBL	≤0.03–0.25	≤0.03	≤0.03	NR	NR
					*E. coli*	61	ESBL-prod.	≤0.03–0.06	≤0.03	≤0.03	NR	NR
					*E. coli*	229	Non-ESBL-prod.	≤0.03–0.25	≤0.03	≤0.03	NR	NR
Mendes [19]	2022	USA	Multi-center	UTIs	*E. coli*	2035	Non-ESBL-prod.	≤0.004–0.25	0.015	0.015	NR	NR
					*E. coli*	360	ESBL-prod.	0.008–4	0.015	0.03	NR	NR
Mendes [20]	2023	USA	Multi-center	UTIs	Enterobacterales	3576	442 ESBL	≤0.004–>8	0.015	0.06	NR	NR
					*E. coli*	2339	351 ESBL	≤0.004–4	0.015	0.015	NR	NR
					*K. pneumoniae*	511	81 ESBL	0.008–>8	0.015	0.03	NR	NR
					*P. mirabilis*	235	10 ESBL	0.008–0.25	0.12	0.12	NR	NR
Ranasinghe [21]	2022	Various ^d^	Multi-center	BSIs	*E. coli*	274	235 ESBL, 24 pAmpC, 4 ESBL and pAmpC	0.015–0.25	0.03	0.03	NR	NR
					*K. pneumoniae*	42	33 ESBL, 2 ESBL and pAmpC, 1 pAmpC	0.025–0.25	0.03	0.125	NR	NR
Yao [1]	2016	China	Multi-center	NR	*E. coli*	85	NR	NR	≤0.125	1	NR	NR
					*K. pneumoniae*	80	NR	NR	≤0.125	0.5	NR	NR
					*P. mirabilis*	15	NR	NR	≤0.125	≤0.125	NR	NR
					*P. aeruginosa*	25	NR	NR	8	64	NR	NR
					*E. cloacae*	50	NR	NR	≤0.125	1	NR	NR
					*Ε* *.aerogenes*	30	NR	NR	≤0.125	≤0.125	NR	NR
					*C. freundii*	25	NR	NR	≤0.125	0.25	NR	NR

Abbreviations: *E. aerogenes*, *Enterobacter aerogenes*; *E. cloacae*, *Enterobacter cloacae*; *E. coli*, *Escherichia coli*; ESBL, extended spectrum β-lactamase; I, intermediate resistance; *K. pneumoniae*, *Klebsiella pneumoniae;* MIC, minimum inhibitory concentration; N, total number of isolates; n, number of isolates; MDR, multidrug-resistant; NR, not reported; pAmpC, plasmid-mediated AmpC β-lactamase; *P. aeruginosa*, *Pseudomonas aeruginosa*; *P. mirabilis*, *Proteus mirabilis*; prod, producing; R, resistance; UTI, urinary tract infection. Notes: ^a^ The countries in Europe were Belgium, France, Germany, Greece, Ireland, Italy, Poland, Portugal, Spain, Sweden, and the United Kingdom; ^b^ Other sources were blood, respiratory tract, and ear drainage; ^c^ Interpretative criteria according to the authors: susceptible MIC ≤ 0.125 mg/L; intermediate MIC = 0.25 mg/L; resistant MIC ≥ 0.5 mg/L; non-susceptible isolates (susceptible isolates were defined by the authors as those with an MIC ≤ 0.125 mg/L); ^d^ Australia, Canada, Italy, Lebanon, New Zealand, Saudi Arabia, Singapore, South Africa, Turkey.

**Table 2:** Resistance of Gram-positive bacterial isolates to tebipenem.

Author	Year	Country	Single or Multicenter (Surveillance)	Sources of Isolation (Infection or Body Site)	Isolates (Subgroup Evaluated)	N	Resistance Mechanism *n* (%)	MIC Range	MIC_50_	MIC_90_
								mg/L		
Gerges [17]	2023	USA	Single-center	Blood cultures	*E. faecalis*	35	Vancomycin-susceptible	≤0.004–>4	0.25	1
					*E. faecium*	35	Vancomycin-susceptible	0.125–>4	1	>4
					*E. faecium*	20	Vancomycin-resistant	4–>4	>4	>4
					Group B β-hemolytic streptococci	12	NR	≤0.004–0.015	≤0.004	0.015
Yao [1]	2016	China	Multi-center	NR	*E. faecalis*	10	NR	NR	0.25	32
					*E. faecium*	10	NR	NR	64	128

Abbreviations: *E. faecalis*, *Enterococcus faecalis*; I, intermediate resistance; MIC, minimum inhibitory concentration; N, total number of isolates; n, number of isolates; NR, not reported; R, resistance.

## Data Availability

No new data were created or analyzed in this study. Data sharing is not applicable to this article.

## Supplementary Materials

The following supporting information can be downloaded at: https://www.mdpi.com/article/10.3390/microorganisms14030726/s1, Table S1: Search strings for each resource, performed on 17 October 2025.

## Abbreviations

AP	acute pyelonephritis
ATP	adenosine triphosphate
cUTI	complicated urinary tract infection
ESBL	extended-spectrum β-lactamase
FDA	U.S. Food and Drug Administration
IMP	imipenemase
KPC	*K. pneumoniae* carbapenemase
MDR	multidrug-resistant
MIC	minimum inhibitory concentration
NDM-1	New Delhi metallo-β-lactamase-1
OXA-48	Oxacillinase-48
PBP	penicillin-binding protein
VIM	Verona integron-encoded metallo-β-lactamase
