# Correction: Falagas et al. Resistance of Uropathogens to Tebipenem: An Analysis of the Evidence from In Vitro Antimicrobial Susceptibility Studies. *Microorganisms* 2026, *14,* 726

**DOI:** 10.3390/microorganisms14092081

**Published:** 2026-09-17

**Authors:** Matthew E. Falagas, Christina-Maria Asimotou, Dimitrios S. Kontogiannis, Laura T. Romanos, Panagiota Poziou, Iva D. Tzvetanova

**Affiliations:** 1 Alfa Institute of Biomedical Sciences, 15123 Athens, Greece; maritinasilver@gmail.com (C.-M.A.); kontogiannisds@gmail.com (D.S.K.); l.romanos@aibs.gr (L.T.R.); peny.poziou@gmail.com (P.P.); 2 School of Medicine, European University Cyprus, 2404 Nicosia, Cyprus; i.tzvetanova@euc.ac.cy; 3 Department of Medicine, Tufts University School of Medicine, Boston, MA 02111, USA

Due to an oversight by the Editorial Office, Figure 1 in the original publication [1] contained a mistake. The correct Figure 1 appears below.

The authors state that the scientific conclusions are unaffected. This correction was approved by the Academic Editor. The original publication has also been updated.

## Figures and Tables

**Figure 1:**
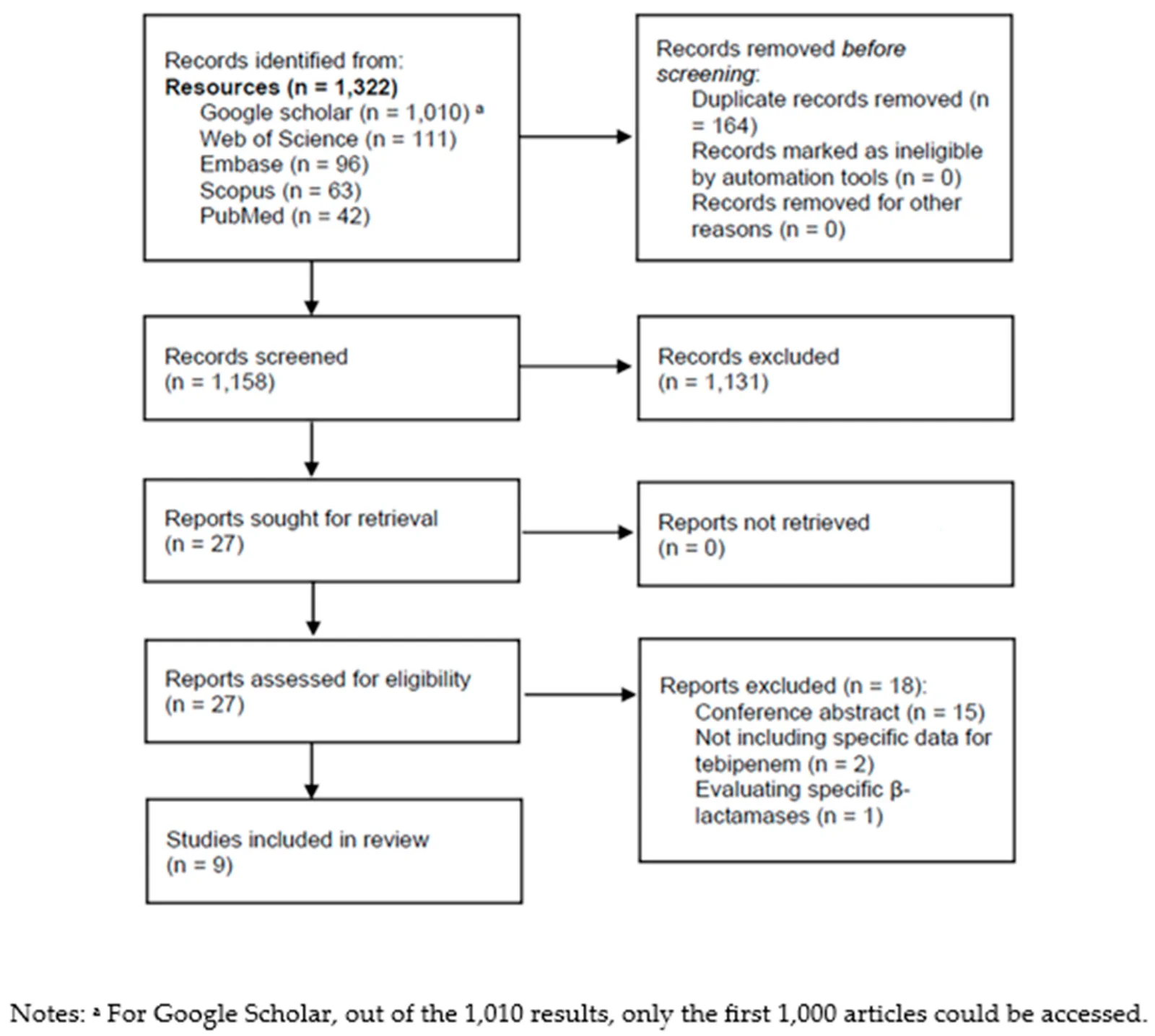
Flow diagram of article selection.
