# Supplementary material for: Resistance of Uropathogens to Tebipenem: An Analysis of the Evidence from In Vitro Antimicrobial Susceptibility Studies
Source: Microorganisms. 2026 Mar 23;14(3):726. doi: 10.3390/microorganisms14030726 (PMC13028607; doi:10.3390/microorganisms14030726)
Supplement: Supplementary file 1 [file microorganisms-14-00726-s001.zip › microorganisms-4153514-supplementary.pdf]

Table S1. Search strings for each resource, performed on 17 October 2025.

Platform	Search String	Coverage (Years)	Results
Google Scholar [a]	(tebipenem OR "SPR994") AND in vitro AND (susceptibility OR resistance OR "antimicrobial susceptibility" OR "antimicrobial resistance" OR MIC OR "minimum inhibitory concentration")	NA	1010
Web of Science	(tebipenem OR "SPR994") AND in vitro AND (susceptibility OR resistance OR "antimicrobial susceptibility" OR "antimicrobial resistance" OR MIC OR "minimum inhibitory concentration")	1900 – 2025	111
Embase	(tebipenem OR "SPR994") AND in vitro AND (susceptibility OR resistance OR "antimicrobial susceptibility" OR "antimicrobial resistance" OR MIC OR "minimum inhibitory concentration")	1974 – 2025	96
Scopus	(tebipenem OR "SPR994") AND in vitro AND (susceptibility OR resistance OR "antimicrobial susceptibility" OR "antimicrobial resistance" OR MIC OR "minimum inhibitory concentration")	1788 – 2025	63
PubMed	(tebipenem OR "SPR994") AND in vitro AND (susceptibility OR resistance OR "antimicrobial susceptibility" OR "antimicrobial resistance" OR MIC OR "minimum inhibitory concentration")	1946 – 2025	42

Notes: [a] For Google Scholar, only the first 1000 articles could be accessed.
